# Metagenome-Assembled Genomes from Amazonian Soil Microbial Consortia

**DOI:** 10.1128/mra.00804-22

**Published:** 2022-10-27

**Authors:** Jéssica A. Mandro, Fernanda M. Nakamura, Júlia B. Gontijo, Siu M. Tsai, Andressa M. Venturini

**Affiliations:** a Cell and Molecular Biology Laboratory, Center for Nuclear Energy in Agriculture, University of São Paulo, Piracicaba, SP, Brazil; b Princeton Institute for International and Regional Studies, Princeton University, Princeton, New Jersey, USA; University of Arizona

## Abstract

Here, we report 17 metagenome-assembled genomes (MAGs) recovered from microbial consortia of forest and pasture soils in the Brazilian Eastern Amazon. The bacterial MAGs have the potential to act in important ecological processes, including carbohydrate degradation and sulfur and nitrogen cycling.

## ANNOUNCEMENT

The soil microbiota is responsible for several ecological functions (1) that drive biogeochemical processes and ecosystem services (2). However, losses of microbial diversity due to global changes can affect these processes (3). The Amazon is the largest reservoir of biodiversity on Earth (4, 5), and changes in land use modify soil microbial communities, leading to alterations in their taxonomic and functional profiles (5–9). Here, we used culture-dependent and -independent techniques to assess the microbial diversity of Amazonian soils. We developed a cultivation experiment using methane (CH_4_) to obtain metagenome-assembled genomes (MAGs) related to the carbon cycle, which resulted in two microbial consortia.

Soil sampling was conducted in the Brazilian Eastern Amazon, state of Pará, in a primary forest located in the Tapajós National Forest (2°51′19.6″S 54°57′30.1″W) and a pasture in the adjacent region (3°07′44.9″S 54°57′15.5″W). In each area, soil samples from 0- to 10-cm depth were collected in quintuplicate after removing the litter layer. Then, the samples of each land use were mixed. Microbial consortia were obtained from 10 g of forest and pasture soil samples which were initially triplicate enriched in CH_4_ atmosphere (12% vol/vol) for 15 days and serial diluted using the roll-tube technique (10) with a mixture (1:1) of nitrate mineral salt (NMS) medium (from the German Collection of Microorganisms and Cell Cultures [DSMZ], https://www.dsmz.de/microorganisms/medium/pdf/DSMZ_Medium632.pdf) without methanol and Bushnell Haas medium (HiMedia Lab Pvt. Ltd., Mumbai, India) under CH_4_ atmosphere (12%, vol/vol). From the microbial enrichment and cultivation in triplicate, we selected one forest and one pasture consortia sample for further analyses. The total consortia DNA was extracted using the PowerLyzer PowerSoil DNA Isolation Kit (Qiagen, Hilden, Germany). The metagenomic libraries were constructed using the Nextera DNA Flex Library Prep Kit (New England BioLabs, Inc., Ipswich, MA) and sequenced on the HiSeq 2500 platform (Illumina Inc., San Diego, CA) (2 × 100 bp).

The 38.5 and 42.1 million paired-end reads from the forest and pasture, respectively, were imported into the KBase platform (11), and default parameters were used for all software unless otherwise specified. Reads were quality evaluated using FastQC v0.11.9 (12) and filtered using Trimmomatic v0.36 (adapters, NexteraPE-PE; quality score, Phred scores >20) (13). After quality control, 35.1 and 38.3 million paired-end reads were maintained for forest and pasture, respectively. Data were assembled using MetaSpades v3.15.3 (14) and then binned with MaxBin2 v2.2.4 (15). The total size of the metagenomic assembly for forest was 46.11 Mbp (contigs, 2,883; GC content, 64.87%; *N*_50_, 56,397 bp) and for pasture was 89.07 Mbp (contigs, 7,503; GC content, 63.91%; *N*_50_, 29,115 bp).

CheckM v2.2.4 (16) was used to determine bin quality. Bins were quality filtered (completeness of ≥50% and contamination of ≤10%), extracted using Extract Bins as Assemblies from BinnedContigs v1.0.2, and taxonomically classified by GTDB-Tk v1.7.0 (R202) (17). We used DRAM v0.1.0 (18) for functional annotation. Bin relative abundance (the number of mapped reads divided by the number of reads in the corresponding metagenome) was calculated using Bowtie2 v2.3.2 (19).

**FIG 1:**
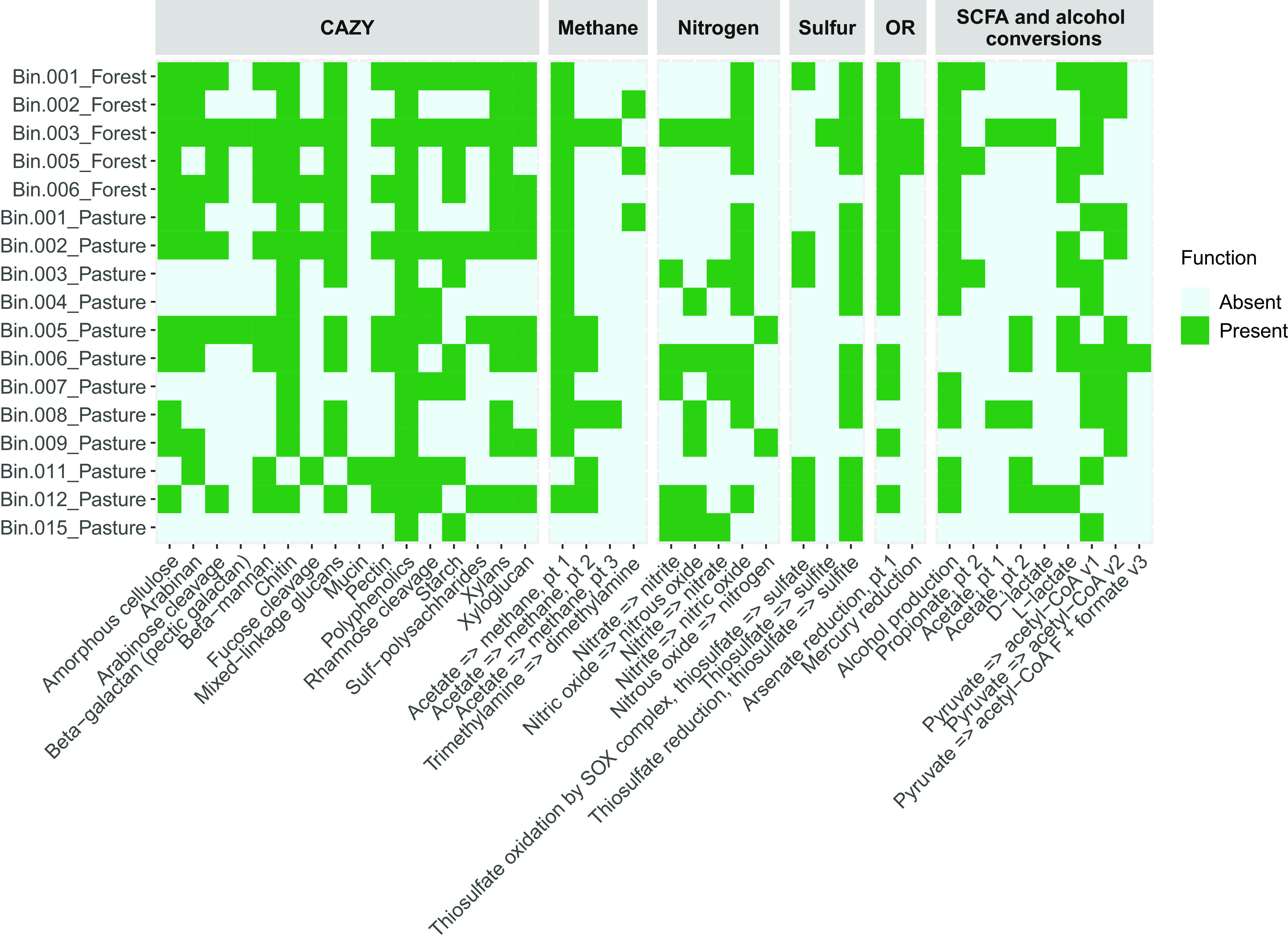
DRAM annotations of the consortia metagenome-assembled genomes (MAGs) from Amazonian forest and pasture soils. The colors in the heatmap represent the presence or absence of relevant metabolic functions in each MAG. CAZy, carbohydrate-active enzymes; OR, other reductases; SCFA, short-chain fatty acids.

We recovered 17 bacterial MAGs, assigned to *Proteobacteria* (11 MAGs), *Actinobacteriota* (4 MAGs), and *Bacteroidota* (2 MAGs) (Table 1). Functional annotations showed carbohydrate-active enzyme genes (CAZymes) and others associated with the biogeochemical cycles of nitrogen, sulfur, and methane (Fig. 1), indicating the potential ecological roles of these organisms.

**TABLE 1:** Features and accession numbers of the consortia metagenome-assembled genomes (MAGs) from Amazonian forest and pasture soils

MAG	GTDB classification	Size (Mb)	No. of contigs	*N*_50_ (bp)	GC content (%)	No. of CDS^*a*^	Coverage (×)	Compl. (%)/cont. (%)^*b*^	Relative abundance (%)^*c*^	GenBank accession no.	SRA accession no.^*d*^
Bin.001_Forest	*Bacteria*; *Proteobacteria*; *Alphaproteobacteria*; *Rhizobiales*; *Xanthobacteraceae*; *62-47*	5.16	107	151,135	61.99	4,859	425	99.58/1.53	39.29	JANINJ000000000	SRR19663812
Bin.002_Forest	*Bacteria*; *Actinobacteriota*; *Actinomycetia*; *Propionibacteriales*; *Nocardioidaceae*; *Nocardioides*; Nocardioides kongjuensis	5.49	12	665,384	72.02	5,301	255	99.22/0.52	25.19	JANINK000000000	SRR19663812
Bin.003_Forest	*Bacteria*; *Proteobacteria*; *Gammaproteobacteria*; *Burkholderiales*; *Burkholderiaceae*; *Massilia*; *Massilia sp001426525*	7.47	67	234,711	65.99	6,507	197	99.92/0.77	26.36	JANINL000000000	SRR19663812
Bin.005_Forest	*Bacteria*; *Proteobacteria*; *Alphaproteobacteria*; *Rhizobiales*; *Rhizobiaceae*; *Mesorhizobium*	6.68	565	20,288	63.77	6,845	30	95.10/4.34	0.34	JANINM000000000	SRR19663812
Bin.006_Forest	*Bacteria*; *Proteobacteria*; *Alphaproteobacteria*; *Sphingomonadales*; *Sphingomonadaceae*; *Sphingomonas*	3.24	63	88,057	68.09	3,102	14	90.91/1.45	0.87	JANINN000000000	SRR19663812
Bin.001_Pasture	*Bacteria*; *Actinobacteriota*; *Actinomycetia*; *Propionibacteriales*; *Nocardioidaceae*; *Nocardioides*; Nocardioides kongjuensis	5.49	19	494,217	72.03	5,305	681	99.22/0.52	61.53	JANINO000000000	SRR19663813
Bin.002_Pasture	*Bacteria*; *Proteobacteria*; *Alphaproteobacteria*; *Sphingomonadales*; *Sphingomonadaceae*; *Sphingomonas*	4.63	8	1,260,393	64.76	4,308	57	99.57/2.32	4.40	JANINP000000000	SRR19663813
Bin.003_Pasture	*Bacteria*; *Proteobacteria*; *Alphaproteobacteria*; *Rhizobiales*; *Xanthobacteraceae*; *Afipia*	4.43	22	649,841	61.44	4,294	47	97.41/4.23	3.48	JANINQ000000000	SRR19663813
Bin.004_Pasture	*Bacteria*; *Proteobacteria*; *Gammaproteobacteria*; *Xanthomonadales*; *Xanthomonadaceae*; *Luteimonas_B*	2.39	157	21,784	71.50	2,401	35	97.30/0.43	1.41	JANINR000000000	SRR19663813
Bin.005_Pasture	*Bacteria*; *Bacteroidota*; *Bacteroidia*; *Chitinophagales*; *Chitinophagaceae*	3.88	8	1,394,606	40.12	3,389	33	99.51/0.49	2.12	JANINS000000000	SRR19663813
Bin.006_Pasture	*Bacteria*; *Actinobacteriota*; *Actinomycetia*; *Propionibacteriales*; *Nocardioidaceae*; *Nocardioides*	4.75	453	19,777	72.07	4,947	50	95.64/2.33	3.92	JANINT000000000	SRR19663813
Bin.007_Pasture	*Bacteria*; *Proteobacteria*; *Alphaproteobacteria*; *Caulobacterales*; *Caulobacteraceae*; *Phenylobacterium*	4.81	359	22,875	69.48	4,961	22	93.79/5.01	1.80	JANINU000000000	SRR19663813
Bin.008_Pasture	*Bacteria*; *Proteobacteria*; *Gammaproteobacteria*; *Xanthomonadales*; *Rhodanobacteraceae*; *Dokdonella_A*; *Dokdonella_A fugitiva_A*	4.68	186	45,676	69.77	4,285	14	98.51/1.29	1.14	JANINV000000000	SRR19663813
Bin.009_Pasture	*Bacteria*; *Bacteroidota*; *Bacteroidia*; *Chitinophagales*; *Chitinophagaceae*; *Flavipsychrobacter*	3.76	98	277,925	43.57	3,588	11	98.52/2.00	0.72	JANINW000000000	SRR19663813
Bin.011_Pasture	*Bacteria*; *Actinobacteriota*; *Actinomycetia*; *Actinomycetales*; *Micrococcaceae*; *Paenarthrobacter*	3.18	956	3,691	62.51	4,297	9	65.99/0.70	0.48	JANINX000000000	SRR19663813
Bin.012_Pasture	*Bacteria*; *Proteobacteria*; *Alphaproteobacteria*; *Rhizobiales*; *Rhizobiaceae*; *Agrobacterium*; Agrobacterium pusense	5.28	388	23,271	57.97	5,743	7	71.63/5.42	0.64	JANINY000000000	SRR19663813
Bin.015_Pasture	*Bacteria*; *Proteobacteria*; *Gammaproteobacteria*; *Burkholderiales*; *Burkholderiaceae*; *Ramlibacter*	4.05	537	10,675	69.32	4,568	6	81.02/1.64	0.45	JANINZ000000000	SRR19663813

a CDS, coding sequences.

b Compl., completeness value; Cont., contamination value.

c Relative abundance, the number of mapped reads divided by the number of reads in the corresponding metagenome.

d SRA accession number of the raw metagenomic reads for each MAG.

### Data availability.

All data are deposited at the NCBI under BioProject PRJNA849167. The SRA accession numbers for the raw reads are SRR19663812 and SRR19663813. The metagenome-assembled genomes are deposited under the GenBank accession numbers listed in Table 1. Data are also available on the KBase platform at https://doi.org/10.25982/116951.133/1878567.
